# Home-Based Care for Children with Serious Illness: Ecological Framework and Research Implications

**DOI:** 10.3390/children9081115

**Published:** 2022-07-26

**Authors:** Jackelyn Y. Boyden, Douglas L. Hill, Gwenn LaRagione, Joanne Wolfe, Chris Feudtner

**Affiliations:** 1 Justin Michael Ingerman Center for Palliative Care, The Children’s Hospital of Philadelphia, Philadelphia, PA 19104, USA; hilld@chop.edu (D.L.H.); laragione@chop.edu (G.L.); feudtner@chop.edu (C.F.); 2 Department of Psychosocial Oncology, Dana-Farber Cancer Institute, Boston, MA 02215, USA; joanne_wolfe@dfci.harvard.edu; 3 Department of Pediatrics, Boston Children’s Hospital, Boston, MA 02115, USA; 4 Division of General Pediatrics, The Perelman School of Medicine at the University of Pennsylvania, Philadelphia, PA 19104, USA

**Keywords:** pediatrics, serious illness, home-based care, ecological framework, multilevel research

## Abstract

Care for U.S. children living with serious illness and their families at home is a complex and patchwork system. Improving home-based care for children and families requires a comprehensive, multilevel approach that accounts for and examines relationships across home environments, communities, and social contexts in which children and families live and receive care. We propose a multilevel conceptual framework, guided by Bronfenbrenner’s ecological model, that conceptualizes the complex system of home-based care into five levels. Levels 1 and 2 contain patient and family characteristics. Level 3 contains factors that influence family health, well-being, and experience with care in the home. Level 4 includes the community, including community groups, schools, and providers. Level 5 includes the broader regional system of care that impacts the care of children and families across communities. Finally, care coordination and care disparities transcend levels, impacting care at each level. A multilevel ecological framework of home-based care for children with serious illness and families can be used in future multilevel research to describe and test hypotheses about aspects of this system of care, as well as to inform interventions across levels to improve patient and family outcomes.

## 1. Introduction

Home is where care usually occurs for children with serious illness, or illnesses, which carry “a high risk of mortality AND either negatively impacts a person’s daily function or quality of life, OR excessively strains their caregivers” [1] that are often medically complex and result in high service needs, high health care use, and severe functional limitations [2]. Beyond the newborn period, these children spend most of their last year of life at home [3], and many die at home [4]. Families provide extraordinary amounts of health care and coordination of services for their children at home [5,6], supported by a complex network of family, friends, community groups, schools, hospital-, community-, and home-based providers, insurers, and policymakers [7,8]. 

Home-based health care for children with serious illness and their families in the U.S. is a notably complicated, fragmented, and patchwork system and may include, but is not limited to, primary, palliative, hospice, other sub-specialty, nursing, behavioral health, educational, and spiritual care [9]. Families face challenges accessing consistent, coordinated, high-quality care across the system, including shortages of providers that are able to support children with medically complex serious illness at home, strained continuity and coordination between providers across care settings, inadequate insurance coverage, and regulations that restrict eligibility, access, and reimbursement [7,8,10,11]. These challenges lead to adverse patient and family outcomes, such as poorly controlled symptoms, delayed hospital discharge, excess hospital use, and poorer health and well-being [12,13,14,15,16]. 

Despite these complex challenges across the system of home-based care, existing studies on the barriers and strategies to improve home-based care for children with serious illness focus on one or two levels of the system, such as the impact of provider knowledge or attitudes on home hospice or palliative care provision [17,18,19,20,21] or the effect of organizational and regulatory factors on care access [10,22,23,24,25,26,27]. Other studies have examined health system-level influences (e.g., care coordination, palliative care services) on patient and family quality of life [28,29,30], mental health [31], concordance between the preferred and actual location of death [32,33,34], and experiences and satisfaction with care [32,35,36,37,38,39]. None of these studies employed a multilevel approach to understanding how variations in home, community, regional, state, and national factors interact and collectively impact care access and provision or patient and family outcomes.

Improving home-based care provided to children with serious illness and their families requires a comprehensive multilevel approach to examine relationships across children’s and families’ home environments, communities, and social contexts in which they live and receive care, and to test hypotheses about, and solutions to, problems impacting the system. While a previous publication reviewed multilevel ecological factors influencing the use of pediatric palliative care in the community, it did not propose a testable framework for how factors interact to affect care outcomes [7]. In this paper, we propose a multilevel ecological conceptual framework for home-based care for children with serious illness. We also describe implications for using this framework in future research to test important hypotheses about how care is provided and to study interventions to improve care outcomes for children with serious illness and their families in homes and across communities. While the following framework focuses on U.S.-based care, the same tenets can likely be applied in other settings.

## 2. Ecological Framework of Human Development

Bronfenbrenner, a child developmental psychologist, put forward an ecological model for studying children’s development within their immediate setting and within their social and environmental contexts (Table 1). Bronfenbrenner called for naturalistic ecological experiments to understand how people and environments adapt to, accommodate, or interact with each other [40]. Controlling as many “theoretically relevant system properties as practical and possible within a study’s design” is imperative in rigorously designed ecological experiments [40] (p. 518). Importantly, Bronfenbrenner’s model accounts for the reciprocal interaction across and within levels of influence; that is, it accounts for the effect of Factor A on B, but also for the potential effect of Factor B on A [40].

**Figure 1:**
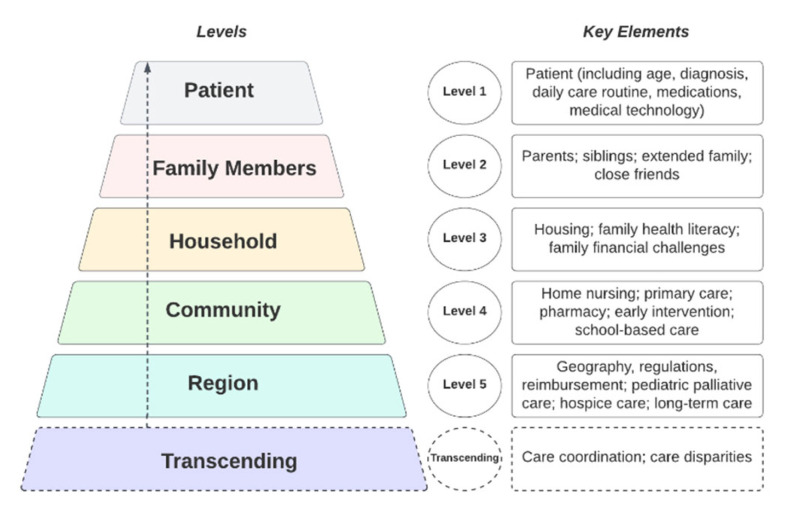
Multilevel ecological framework of home-based care for children with serious illness and their families. Created in Lucidchart (www.lucidchart.com accessed on 21 July 2022).

Drawing on Bronfenbrenner’s model and our own interdisciplinary clinical and research experience, based on an extensive review of the literature and building on our previous papers that describe the facilitators and preferences for high-quality pediatric palliative care at home [7,41], we developed the following framework to conceptualize the complex system of home-based care that supports children with serious illness and their families. This framework (Figure 1), which consists of five levels spanning the patient-level to the regional-level of care, was developed under the following assumptions:

(1) We organized as many key elements of the system of home-based care into each level as possible, but we acknowledge that the elements within each level are not exhaustive;
(2) We organized these elements into levels according to where each element *originates*, rather than by where the effect of each element is *felt*. To give an example, home care nursing, located at the community level, is provided by community agencies, although the effect of home care nursing is felt at the individual patient and family level;
(3) While we organized these elements systematically and with careful consideration, we acknowledge that the levels of the actual system of home-based care may overlap in complex ways and the individual elements are somewhat fluid; that is, the individual elements could fit, conceptually, in other levels based on individual circumstances and under specific social, economic, and geographic factors;
(4) The proposed framework serves as a starting point for building future research studies and may be modified as knowledge is generated from future studies.

## 3. Conceptual Framework of Home-Based Care for Children with Serious Illness and Their Families

### 3.1. Level 1—Patient

Patients living with serious illness who receive home-based care include children with advanced cancer, as well as a range of complex chronic conditions (CCCs) [42,43,44]. With medical advances, more of these children survive longer with increasing medical complexity, medical technology dependency, and symptom burden [45,46,47,48]. These children’s symptoms and quality of life are directly affected by their daily care routines, including medications and supportive medical, mobility, and communication technologies. Care routines in turn are directly informed by children’s symptoms and quality of life [30,33]. We also include patient demographic and clinical characteristics (e.g., a child’s actual and developmental ages, diagnosis) and goals of care at this level, although they are often generated or influenced by parents, since they directly impact the care patients receive.

### 3.2. Level 2—Family Members

This level encompasses individual members of the patient’s “family,” which could include parents or other primary caregivers, siblings, extended family and even close friends who support patients and their families. Parents provide the majority of care [6], although siblings also play important roles [16,49]. While the family impacts the child’s care, a child’s illness also impacts the physical and mental health and well-being of family members [50,51,52,53,54]. Negative impacts can be mitigated by extended family and close friends [55], who can provide emotional support and help with tasks such as cooking, cleaning, and childcare [56,57].

### 3.3. Level 3—Household

This level encompasses some of the social determinants of health that may affect family health, well-being, and care experiences at the household level, rather than at the level of the individual family members.

#### 3.3.1. Housing

The health care needs of children with serious illness may impact families’ housing preferences (including the safety and accessibility of the home environment), as well as housing possibilities (availability of housing in a given community or neighborhood that is able to support children’s care needs) [58,59]. Additionally, housing characteristics (such as home safety and accessibility) also impact the care patients receive and the physical and mental outcomes of patients and family members [58,59]. 

#### 3.3.2. Family Health Literacy and Language Proficiency

People with low health literacy (specifically, lower ability to: read, comprehend, and interpret written language; use and interpret quantitative information; listen and speak effectively) generally have a lower ability to understand and adhere to medical advice, poorer use of health services, and poorer health outcomes [60]. For example, families with limited English proficiency face additional barriers in navigating the health care system, accessing home-based services (e.g., hospice, pharmacy, home care), and understanding instructions or making decisions for their child’s illness [61,62,63,64]. 

#### 3.3.3. Family Financial Challenges

Families of children with serious illness may experience financial difficulty, household material hardships (including difficulty paying for rent, energy bills, and food), unstable or unsafe living conditions, as well as under- or un-insurance that negatively impact children’s and families’ wellbeing [53,57] and further reduce access to critical home-based services (e.g., home nursing, home hospice, pharmacies) [32,57,64,65,66,67,68]. Children with serious illness in low-income households may experience higher symptom burdens and a lower quality of life compared to children in high-income families [69].

Parents also report struggling with medical expenses and lost income due to missed work [32,57,66,69,70,71]; finding employment that accommodates the time demands of caring for children with serious illness [72]; loss of employment as a result of their children’s medical needs [32,57,67,73], increasing stress and decreasing parental well-being. 

### 3.4. Level 4—Community

Patients’ and families’ experiences are affected by community groups, schools, hospital- and community-based providers, and other non-medical services, including transportation, pharmacy, infusion, medical supply services, respite care, counseling and bereavement care, and spiritual, religious, and cultural organizations. To distinguish from the regional level below, we focus here on factors families encounter as they seek care in their immediate community. This level of care is particularly extensive, and we highlight only a few key elements.

#### 3.4.1. Home Nursing Services

Home health nursing (HHN) provides physical assessment, respiratory care, wound care, symptom management, medication management, respite care, and other support to children at home [39,74]. While HHN is associated with decreased hospitalizations among patients [75] and higher levels of parental well-being [13,76], the availability of HHN sufficiently skilled, competent, and comfortable caring for children with serious illness is limited [57,67,77,78,79,80]. As high as eighty percent of parents report unfilled HHN hours or missed shifts due to inadequate staffing, absenteeism, and turnover [57,67,80,81], which is associated with delayed hospital discharge [14,67,82], excess hospital use [75,83], higher readmission rates [15,75], reduced parental employment [57], and poorer parental well-being [12,13,57,67,80]. Regulations, funding, and reimbursement (described later) may also facilitate or impede families’ access to adequate home nursing services [80].

#### 3.4.2. Primary Care

While some children with serious illness primarily receive care from a specialty team (such as oncology, cardiology, and complex care), the main source of medical management in the community for many children is their primary care physician (PCP) in their medical home [84]. PCPs may work closely with children’s other providers and be involved in hospital discharge planning, medical decision-making, coordinating home nursing and equipment providers, and communicating and coordinating with educational services [39,85,86]. A close partnership between hospital-based providers and PCPs may reduce hospitalizations and costs [87] and increase parental satisfaction with care [88]. A PCPs involvement has also been associated with increased use of hospice or home health care in the last year of life [89]. 

#### 3.4.3. Pharmacy

Parents of children with CCCs report significant unmet needs for prescription medications [90], which could be associated with factors such as inadequate access to transportation or lack of access to pharmacies (i.e., “pharmacy deserts”), particularly in underserved communities [91,92]. 

#### 3.4.4. Early Intervention

Infants and toddlers with serious illness may benefit from early intervention (EI) programs to reduce the impact of disability, prevent further complications, and promote optimal health, function, and quality of life [93,94]. Some EI providers, however, express discomfort with working with children with CCCs and report needing additional training [94]. Other studies have documented challenges with EI program funding, eligibility criteria, referrals, and coordination of services [95,96]. 

#### 3.4.5. School-Based Care

Children with serious illness may benefit from a formal school environment. Facilitating school attendance for these children requires extensive communication, coordination, and collaboration between families, educators, school nurses, private duty nurses, PCPs, home-based providers, and specialty providers [97,98]. Parents report frustrations and challenges communicating with schools and coordinating services across schools and medical providers [99]. Some families may have limited relationships with school nurses due to staffing shortages or limited school nurse training and experience in working with children with CCCs [99,100]. Some families may not be able to fill the nursing hours required by their children to safely attend school [67].

### 3.5. Level 5—Region

The community and regional levels overlap, although several key elements of the broader systems of regionalized care directly affect children and families across communities.

#### 3.5.1. Geography, Regulations, and Reimbursement

Access to home-based services for children with serious illness depends on many factors, such as where the family resides in relation to metropolises with children’s hospitals [22,101,102]. Families who live significant distances from children’s hospitals face additional challenges, including long ambulance or car trips in the event of crises that local hospital emergency departments are not equipped to handle. Families living in rural areas or lower-income communities also have limited home-based care options [25]. 

Care is also impacted by state- and federal-level regulatory and funding factors, such as concurrent care, waiver programs, hospice eligibility criteria, certificate of need regulations, and the Centers for Medicare and Medicaid certification and reimbursement [7,11,26,57]. These regulations and funding arrangements differ by state or region, resulting in variation in how local care systems are organized and how care is delivered to and accessed by children with serious illness and their families [24,26,57,103,104]. 

#### 3.5.2. Pediatric Palliative Care (PPC)

The total number of PPC organizations and providers across the U.S. caring for children at home is unknown, although only approximately 30% of hospital-based PPC programs in the U.S. offer home visitation services [104,105]. Few studies have systematically evaluated outcomes from these programs, outside of a handful of single-institution program evaluations, which observed improved child quality of life [33,37]; decreased family stress [31]; improved parental quality of life [30]; improved family satisfaction with care [36,106]; improved concordance between the preferred and actual location of death [33,34,106]; and reduced hospital utilization and costs [30,107]. Parents of children who die at home with palliative care support may experience better psychological and bereavement outcomes than parents of children who die in the hospital [108,109].

#### 3.5.3. Hospice Care

A few studies of pediatric hospice care in the U.S. have found that care is often provided by adult hospice providers who lack sufficient pediatric training or experience [17,20]. While one study found that parents were generally satisfied with their child’s symptom management provided by hospice [110], others noted significant problems with scheduling, staffing, and symptom management, resulting in unplanned hospital readmissions [111,112]. Telehealth hospice models may reduce the gap in pediatric-trained hospice providers and improve the end-of-life care of children at home [25].

#### 3.5.4. Long-Term Care (LTC)

A small subset of children with serious illness receive care in residential facilities, including skilled nursing facilities, intermediate care facilities, specialty hospitals that provide LTC, residential schools, and medical group homes [113]; these serve as the children’s main residences. One study of children who died in LTC found that parents were satisfied with their child’s end-of-life care [114], and parents of children receiving LTC were found to have better reported physical health and family functioning when compared to parents of children receiving home care or medical day care [12]. LTC facilities, though, are also challenged by staffing shortages [114], limited funding [113], insufficient end-of-life care policies, and inadequate staff training [115].

### 3.6. Transcending Levels

At least two elements of this framework are transcending; that is, they originate from more than one level of the system of home-based care. 

#### 3.6.1. Care Coordination

Families caring for children with serious illness often shoulder the primary responsibility for arranging and coordinating the extensive healthcare, educational, and social services across the levels of the home-based care system [116,117], as described above. While well-coordinated care has been identified by families as an integral component of high-quality home-based care for children with serious illness [41], families often describe poorly coordinated services [81,90,116,117], leading to negative child and family outcomes [81,116], unplanned hospitalizations [116], and strained family relationships [116,117]. 

In this conceptual framework, care coordination spans across levels, attempting to functionally integrate different system levels to serve patients’ and families’ day-to-day needs. Care coordination includes communication and co-management of care between family and providers; facilitation of family education; support for care transitions; development of written care plans that integrate information from multiple providers and includes patient- and family-centered goals [116].

Often described [103,118] and prescribed [119,120] as an essential element of care for children with CCCs, studies of the impact of care coordination have consistently shown greater parental satisfaction, but mixed findings for patient outcomes and care utilization [121,122,123,124]. This may be attributed, in part, to ambiguity regarding which team, if any, is responsible for care coordination (e.g., home nursing, palliative care, primary care, or sub-specialty teams such as oncology or complex care). 

#### 3.6.2. Care Disparities

Families who experience discrimination based on race, ethnicity, or socioeconomic status at multiple levels of the system may have reduced access to high-quality care, be more likely to experience communication problems and conflict with providers, and be more likely to experience toxic stress [125,126,127], which may interfere with efforts to obtain and coordinate home-based care and lead to poorer patient and family outcomes. Non-Hispanic White children with cancer appear to spend less time in the hospital during the last days of life compared with non-White children, which could be related to medical necessity or parental preference, or to other structural factors, including geographic location, home environment, safety, trust in medical care, and quality of family-provider communication [128]. A study on adult hospice patients found that differences in medical intensity at the end of life (i.e., hospital admissions, emergency department visits, and hospice disenrollment) may be associated with patients’ race, rather than with hospice-level variation in care [129]; children’s race and ethnicity may similarly impact hospice enrollment [130].

## 4. Research Implications

Our multilevel ecological framework provides an organizing schema for investigating patient, family, home, community, and regional factors that affect patient and family outcomes, as well as factors that interact with or modify those effects. This framework could serve as “scaffolding” for research question development, study design, and data collection and analysis [131]. Since these ecological structures, and the ‘processes taking place within and between them’ [40] (p. 518), are interdependent and, therefore, should be analyzed in systems terms, this framework is particularly well-suited for multilevel observational and intervention studies.

### 4.1. Multilevel Observational Studies

Multilevel research methods are compatible with both quantitative and qualitative ecological research designs.

#### 4.1.1. Multilevel Quantitative Research

Multilevel modeling methods provide tools to examine the effects of a factor at one level while controlling for potential confounding at another level, or to examine the potential interactions among factors across levels [131]. This type of research requires clear conceptual models of etiological factors across multiple levels, collection of data from multiple sources, and advanced statistical methods to account for relationships among the levels of analysis, including clustered data across sites, groups, or observations [131]. Additional challenges include collecting higher-level (e.g., neighborhood- and community-level) data and larger sample size requirements needed to detect effects [131]. The complexity of these methodologies has likely contributed to the paucity of studies to test the associations and interactions across multiple levels of the system of care for children with serious illness and their families. Despite these challenges, the potential benefits of exploring these associations and interactions in multilevel statistical models are significant.

Previous studies in adult palliative care have examined patients, the health system, and community factors associated with palliative care registration [132], as well as hospice utilization and disenrollment [129]. Studies outside of palliative care have looked at individual, country, and cultural factors associated with individual health and wellbeing [133,134]. Similarly, a multilevel framework of home-based care for children with serious illness can help us examine the interplay of individual, family, community, and regional influences on care utilization and patient and family outcomes, and the potential mediating roles of effective care coordination and care disparities in this relationship. 

For example, effective care coordination can be hypothesized to mitigate some of the effects of care disparities on patient and family outcomes. Enhanced care coordination may improve access to critical medical and social services in the home and community [116], which could better support children and help parents maintain their employment, improving financial stability [6,73]. This could in turn mitigate the detrimental effects of economic instability on the health and well-being of families who already struggle with the enormous stressors of caring for a child with serious illness [53]. Collectively, these factors could improve child and family outcomes, but multilevel analyses are needed to understand the interplay between care coordination, care disparities, and patient, family, health system, and community factors. 

#### 4.1.2. Multilevel Qualitative Research

Multilevel qualitative approaches have also been used to describe complex phenomena in palliative care, such as caregiver experiences [135], communication barriers [136], and improving care for underserved populations [137]. 

Qualitative approaches can be used to better understand multilevel challenges and explore potential strategies for well-coordinated home-based care from the perspective of patients with serious illness, families, providers, administrators, funders, and policymakers [138].

### 4.2. Multilevel Intervention Studies

Increasingly, determinants of health care disparities and inequities are understood to occur not in isolation [139,140], but rather as “interacting distal, intermediate, and proximal ecological factors” [140] (p. 3). Accordingly, typical health care interventions operating at only one level are insufficient for eliminating health inequities [140]; the goal must be to effect change both within and between different ecological levels. Multilevel interventions, or interventions with components that occur simultaneously or in close succession at multiple levels of influence, are more likely to have a tangible impact on child and family outcomes [131,140] and are vital for eliminating or reducing health disparities [140,141]. 

Methodologically, studies of multilevel interventions need to have a comprehensive guiding theoretical framework or program theory; rigorous study design; appropriate and clearly defined outcomes and measures; strong intervention components; and robust statistical and analytic approaches [142,143]. Few such studies have been performed regarding home-based care for children with serious illness, aside from a multilayered intervention aimed at advocacy, staff capacity, service delivery, and international and regional partnerships to improve general palliative care access and delivery in Sub-Saharan Africa [144]. Similar intervention studies, using the process below, should be implemented to improve access to, and provision of, home-based care for children and families. 

**Figure 2:**
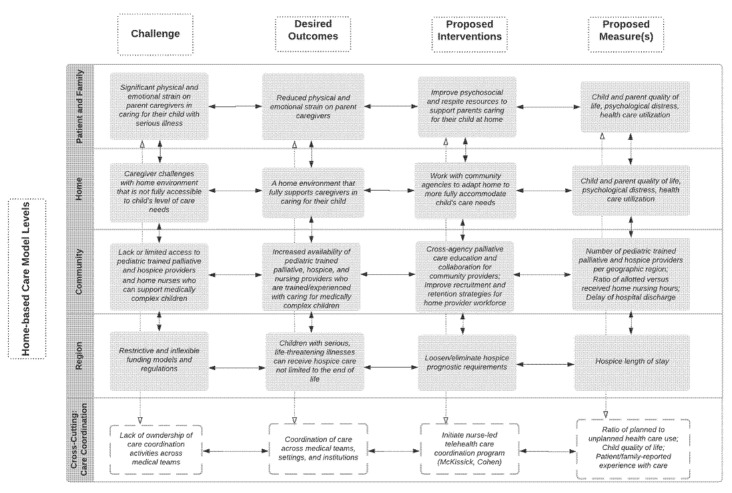
Example multilevel intervention model for home-based care for children with serious illness and their families. This intervention model is derived from the proposed ecological framework (Figure 1). As can be seen, interventions should target all levels of the system of home-based care, including improving the support and strengthening the capacity of family caregivers [31,145]; targeting the capacity of health systems to support children and families in the home through increasing education, training, recruitment, and retention of community-based providers who care for children with serious illness; challenging the existing systems of regulation and financing of care (e.g., loosening or eliminating hospice prognostic requirements) [7,10]; improving care collaboration and coordination between community- and hospital-based institutions [7,28,67,146]; targeting care coordination to bridge interventions across levels. Desired outcomes, specific measures, and analytic strategies should be carefully matched to individual interventions, as well as across levels of intervention [142,143]. Created in Lucidchart (www.lucidchart.com accessed on 21 July 2022).

We begin with the home-based care framework for children with serious illness (Figure 1), which can guide study design, outcome measure selection, and intervention development (Figure 2) [142,143]. In terms of study design, given the focus on the home and community setting and the heterogeneous yet numerically small population of children requiring home-based care, randomized controlled trials may not be feasible; rather, multi-site, natural experiments or time series designs may be more appropriate [142]. In order to maximize the likelihood of intervention success outside of the research context, studies should also involve, at each phase of the study, community stakeholders who provide or receive home-based care for these children and families, including hospital-based providers, community-based providers, policymakers, insurers, schools, and patients and families [143]. 

The framework (Figure 1), and previous research, clinical experience, or stakeholder priorities, can also guide the selection of desired outcomes (e.g., increased availability of home-based providers, reduced strain on parent caregivers), proposed measures (e.g., number of available providers per geographic area, a measure of parental distress), and timing of measurement (Figure 2) [142,143]. These measures may evaluate changes to a system, as well as changes to patients and family members, and may already be in use or may need to be adapted or developed [143]. Selecting appropriate statistical and mixed-methods analytic approaches is critical for understanding outcomes from multilevel intervention studies [142]. While analytic strategies are not represented in Figure 2, they should be carefully matched to individual interventions and across levels of intervention.

Researchers should then develop or adapt, and then refine, interventions [142,143]. This process may require iterative pilot testing and evaluation of intervention acceptability, feasibility, efficacy, and fidelity, particularly if interventions are being adapted to new settings or to specific patient subgroups [142,143]. Interventions at the level of home-based services may target hospice regulations, home nursing recruitment and retention, family respite support, and, importantly, concomitant cross-cutting care coordination (Figure 2). The assessment of interactions between these interventions at multiple levels is also critical to understanding if interventions may impede or augment one another [142]. 

Finally, implementation of the intervention beyond the research study should be considered throughout this process, such as intervention fidelity, reach and uptake, scalability, cost-effectiveness, and sustainability [142,143]. The consideration of these implementation outcomes within the dynamic and multi-dimensional context that may support or hinder intervention impact, including the geographic, organizational, cultural, sociopolitical, economic, and public health contexts, is critical to the long-term success of interventions focused on improving home-based care for children and families [143].

## 5. Conclusions

A multilevel ecological framework of home-based care for children with serious illness and their families can be used in future studies to describe and test hypotheses about aspects of this system of care, as well as to inform interventions to improve patient and family outcomes. 

Challenges will arise in designing and analyzing multilevel studies in this setting. Nevertheless, multilevel ecological studies are not only “worth doing, but…they may be most effective” [141] (p. 1433) for advancing home-based care for children with serious illness and their families, increasing the chances of effective and sustained improvements to the system that can improve patient and family outcomes and impact care disparities for future generations.

## Figures and Tables

**Table 1:** Key Terms and Definitions—Bronfenbrenner’s Ecological Model [40].

Key Term	Definition
Ecological environment	Nested arrangement of structures that are “each contained within the next”
Microsystem	“Complex of relations” between an individual and the environment that exist in that individual’s immediate setting, as defined by place, time, physical features, activity, participant, and role
Mesosystem	“Interrelations” among the major settings in which an individual is situated at a particular point in that individual’s life; in other words, a mesosystem is “a system of microsystems”
Exosystem	An extension of the mesosystem; encompasses other formal or informal social structures that do not directly contain the individual, but that “impinge upon or encompass the immediate settings in which that [individual] is found, and thereby influence, delimit, or even determine what goes on there”; this may include work, neighborhood, mass media, government agencies, etc.
Macrosystem	“Overarching institutional patterns of the culture or subculture” (that is, economic, social, educational, legal, and political systems) that encompass the micro-, meso-, and exo-systems
